# Isolated Hypoglossal Nerve Palsy due to Infected Impacted Tooth

**DOI:** 10.1155/2009/231947

**Published:** 2009-09-14

**Authors:** Farhan Durrani, Royana Singh

**Affiliations:** ^1^Department of Periodontics and Oral Surgery, Faculty of Dental Sciences, Institute of Medical Sciences, Banaras Hindu University, Varanasi, Uttar Pradesh, India; ^2^Department of Anatomy, Institute of Medical Sciences, Banaras Hindu University, Varanasi 221005, India

## Abstract

Case of isolated hypoglossal nerve palsy is an extremely rare condition. There are several causes that can be attributed to it. We present a case where a patient presented herself with swelling on the right side of her cheek extending to the floor of the mouth, with unilateral right hypoglossal nerve palsy. Removal of the impacted tooth resulted in the improvement of function of the hypoglossal nerve. The transient isolated hypoglossal nerve palsy could have been due to the infected impacted tooth. Therefore, the dentist or doctors coming across with isolated hypoglossal nerve palsy should consider the infected impacted tooth as the differential diagnosis.

## 1. Introduction

Isolated hypoglossal nerve palsy is a very rare condition. Several causes are attributed to its aetiology, like, intracranial or extracranial space occupying lesions, head and neck injury, vascular injury, infection, neuropathy or autoimmune disease, or idiopathic [1]. The cases of hypoglossal nerve palsy following impacted tooth extraction have also been reported [2–4]. This paper focuses on an interesting case of transient hypoglossal nerve palsy caused by infected impacted tooth.

## 2. Case Report

**Figure 1:**
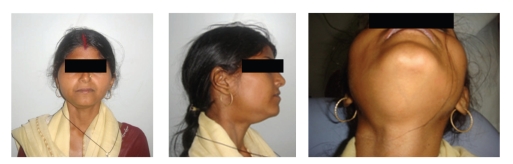
23-year-old female, exhibiting a swelling involving the entire right side of the cheek and floor of the mouth.

**Figure 2:**
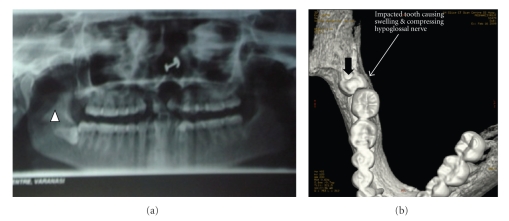
(a) Oral Pantogram showing the impacted third molar on the right lower jaw (arrow head). (b) The denta scan of the lower jaw showing the horizontal lying impacted third molar (arrow).

**Figure 3:**
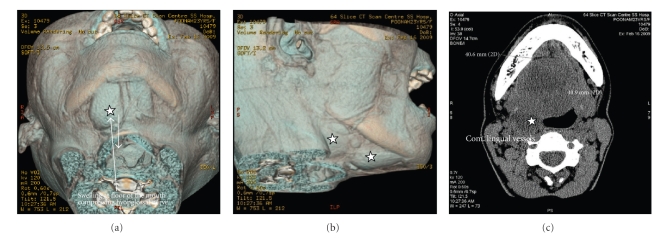
(a) and (b) CT scan of the soft tissue showing a diffused swelling involving the carotid and digastric triangle on the right side (∗). (c) The swelling extending into the retropharyngeal space on the right side (∗).

**Figure 4:**
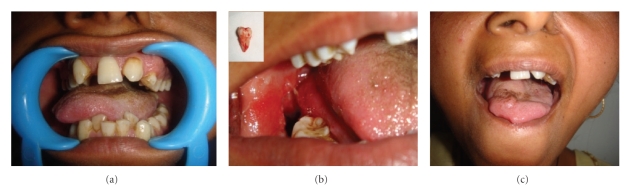
(a) Inability to protrude the tongue and on effort protrusion, the tongue deviated to the right. (b) Impacted third molar extracted. (c) Improvement within two weeks of extraction.

A 23-year-old female attended the OPD, Faculty of Dental Science, with the chief complaint of swelling in the floor of the mouth, difficulty in chewing, and inability to move the tongue and open her mouth fully. She also complained of malaise, pain in the floor of the mouth loss of appetite and loss of weight. She had observed the swelling about 6 months ago with the subsequent difficulty in moving her tongue. There was no history of trauma or illness preceding the swelling. No alteration in the taste or paraesthesia was complained. No relevant medical history was present. On examination, the patient appeared ill looking. Extraoral scarring was not present. A diffused smooth swelling extending from right cheek to the floor of the mouth, obliterating the angle of the mandible, was noted. Asymmetry of the face was very conspicuous (Figure 1). Enlarged lymph nodes were palpated in the submandibular region. On examination of the mouth cavity, the diffused swelling occupied the floor of the mouth on the right side; the oedema of the lower right gingiva and vestibule of the lower jaw were also observed. The tongue was coated, and constant fibrillation of the right side of the tongue was observed. She felt extreme difficulty in protruding her tongue, and on extreme effort to protrude the tongue, it showed deviation to the right side, almost to a right angle (Figure 4(a)). Examination of other cranial nerves was found to be normal and intact. Routine examination of the total count, differential leucocytic count, and immunological and serological examination showed no viral infection or autoimmune disease. Blood glucose and the liver function test were normal. The chest X-ray was clear with no lung pathology. An oral pantogram and CT scan of head and neck were advised to evaluate the possible cause of the hypoglossal nerve palsy. The radiograph and the CT scan revealed the right third molar tooth impacted lying horizontally, deep within the mandibular bone (Figure 2). A diffused swelling confined to the right side of the floor of the mouth, which extended into the oropharynx and retropharyngeal space, was observed (Figures 3(a), 3(b), and 3(c)). No abnormality of the hypoglossal nerve or its sheath itself could be seen in CT scan. The impacted tooth was extracted (Figure 4, Binbox). The patient was put on multivitamins, analgesics, and antibiotics. After two weeks the patient showed tremendous recovery in the hypoglossal nerve function, not only the tongue deviation to the right had lessened but also she could push it to the left side too (Figure 4(c)).

## 3. Discussion

The hypoglossal nerve's carotid space segment, after emerging from the hypoglossal canal, lies deep to the internal carotid artery, the internal jugular vein, the glossopharyngeal nerve, and the vagus nerve. Thereafter, it lies in between the internal jugular vein and the internal carotid artery. The lingual segment of the hypoglossal nerve, at the angle of the mandible, loops around the occipital artery to lie superficially, and at the level of the hyoid bone it lies over the hyoglossus after crossing the lingual vessels, in the sublingual space [5]. Pathology along the carotid space segment or the lingual segment will result in isolated nerve palsy. The investigations done in the present case clearly rule out the intracranial lesions, neuroma or schwannoma of the hypoglossal nerve [6, 7], vascular anomaly of the vertebral or basilar artery [6], massive traumatic haematoma in the deep spaces [8], occipital condyle fracture [9] or following extraction of third molar [3, 10], or head and neck injury [4]. The differential diagnosis included neoplasia, trauma, infection, endocrine, autoimmune, neurologic, and vascular causes [2]. Transient hypoglossal nerve palsy with no etiology has been reported [11]. The probable cause after exclusion in the present case seems to be the infected impacted tooth which may have led to the massive swelling involving not only the floor of the mouth but also the deep pharyngeal space, and thus causing compression of the hypoglossal nerve with the subsequel of transient palsy on the right side. Many causes have, therefore, been attributed to the aetiology of the unilateral isolated hypoglossal nerve palsy; the present case, however, reveals yet another cause of hypoglossal nerve palsy which is infected impacted tooth. The diagnosis is confirmed from the CT scan.

 Freedman et al. [2] has very rightly proposed that isolated Hypoglossal nerve palsy is rare but should be regarded with suspicion. A systematic approach in dealing with this problem should be adopted by the dentist and the doctors to exclude any serious underlying pathology.

## References

[1] Ho MWS, Fardy MJ, Crean StJV (2004). Persistent idiopathic unilateral isolated hypoglossal nerve palsy: a case report. *British Dental Journal*.

[2] Freedman M, Jayasundara H, Stassen LFA (2008). Idiopathic isolated unilateral hypoglossal nerve palsy: a diagnosis of exclusion. *Oral Surgery, Oral Medicine, Oral Pathology, Oral Radiology and Endodontology*.

[3] Dearing J (1998). Transient contralateral hypoglossal nerve palsy following third molar surgery under day-case general anaesthesia: a case report and review of the literature. *British Journal of Oral and Maxillofacial Surgery*.

[4] Omura S, Nakajima Y, Kobayashi S, Ono S, Fujita K (1997). Oral manifestations and differential diagnosis of isolated hypoglossal nerve palsy: report of two cases. *Oral Surgery, Oral Medicine, Oral Pathology, Oral Radiology, and Endodontics*.

[5] Thompson EO, Smoker WR (1994). Hypoglossal nerve palsy: a segmental approach. *Radiographics*.

[6] Graham RM, Thomson EF, Baldwin AJ (2007). Isolated hypoglossal nerve palsy due to a vascular anomaly. *International Journal of Oral and Maxillofacial Surgery*.

[7] Manfredi M, Merigo E, Pavesi G, Macaluso GM, Vescovi P (2007). Tongue lesions and isolated hypoglossal nerve palsy: a case report. *Oral Surgery, Oral Medicine, Oral Pathology, Oral Radiology and Endodontology*.

[8] Vito KJ, Wanamaker JR, Shields RW (1995). Massive hematoma resulting in bilateral hypoglossal nerve paralysis. *Otolaryngology*.

[9] Castling B, Hicks K (1995). Traumatic isolated unilateral hypoglossal nerve palsy: case report and review of the literature. *British Journal of Oral and Maxillofacial Surgery*.

[10] Stankiewicz JA, Pazevic JP (1988). Hypoglossal nerve palsy after tooth extraction. *Journal of Oral and Maxillofacial Surgery*.

[11] Lee SS, Wang SJ, Fuh JL, Liu HC (1994). Transient unilateral hypoglossal nerve palsy: a case report. *Clinical Neurology and Neurosurgery*.

